# Global research on patient involvement in health technology assessment: a bibliometric analysis

**DOI:** 10.1186/s41256-025-00431-z

**Published:** 2025-07-25

**Authors:** Chunlu Yu, Yan Huang, Huamei Wu, Luying Zhang

**Affiliations:** 1 School of Public Health, Fudan University, Shanghai, 200032 China; 2 Center for Chinese Public Administration Research, School of Government, Sun Yat-sen University, Guangzhou, 510275 China; 3 Shanghai Urban Construction Vocational College, Shanghai, 200438 China

**Keywords:** Patient involvement, Health technology assessment, Bibliometric analysis, VOSviewer, CiteSpace

## Abstract

**Background:**

Patient involvement in health technology assessment (HTA) has been extensively explored and implemented in high-income countries, but little is known about it in low- and middle-income countries (LMICs). This study aimed to provide a comprehensive picture of the current state and trends of patient involvement in HTA research, which can inform future research in the LMICs.

**Methods:**

Publications on patient involvement in HTA from January 1, 1900, to December 31, 2023, were retrieved from the core databases of the Web of Science. We applied a bibliometric analysis to reveal the collaboration patterns, hot topics, and evolution of the research field. Co-occurrence, clustering, citation, and burst analyses were performed using VOSviewer and CiteSpace, with results visualized for interpretation.

**Results:**

A total of 175 articles were eligible for inclusion. The first valid article was published in 2000. The number of publications has increased since 2011. The most productive countries and institutions were Canada and McMaster University. The studies focused on five hot topics: patient preferences, priority setting, qualitative research, drug development, and hospital-based HTA. The burst analysis revealed that priority setting and cost effectiveness were the research frontiers.

**Conclusions:**

While patient involvement in HTA research has gained increasing attention, the research conducted in the LMICs remain limited. It is recommended that LMICs participate in international research collaborations, and focus on the five hot topics and emerging frontiers to advance both their research capacity and practical implementations.

## Introduction

Health technology assessment (HTA) is a multidisciplinary process that involves evaluating the clinical, economic, organizational, social, legal, and ethical impacts, as well as the broader implications for patients, relatives, and caregivers of health technologies, by comparing them to available alternatives [1]. It is recognized by the World Health Organization as an important tool for promoting universal health coverage [2]. In the last twenty years, HTA-related academic research has increased rapidly [3], and many high-income countries have integrated HTA into policy practices [4]. More recently, some low- and middle-income countries (LMICs) have also established formal HTA processes to support decision-making on national drug reimbursement lists, the pricing and reimbursement of technologies, and the development of health benefit packages [5–7].

In line with the growing recognition of patient-centered care, HTA agencies and researchers have reached a consensus on the importance of patient involvement in HTA [8]. On the one hand, patients are current or prospective users of health technologies, and are likely to possess valuable empirical knowledge and unique perspectives about their conditions and treatments [9]. Incorporating patient needs, preferences, and experiences into HTA may complement assessments, and help better understand the true value of health technologies for patients [8, 10]. On the other hand, patient involvement enhances transparency of the HTA process, leading to better decisions on the appropriate place for technologies in health care [11]. As HTA becomes increasingly integral to health care reimbursement decision-making, which directly impacts patient benefits, patients have shown great interest in this initiative [12].

Patient involvement is more prevalent in high-income countries where the HTA system is more developed [8]. In these countries, patient involvement is typically institutionalized, with the establishment of laws, action frameworks, and guidelines to guide the practice [13]. For example, in England, the National Institute for Health and Care Excellence (NICE) encourages and solicits patients in different stages of the HTA process. Patients can join consultations to determine scoping areas at the beginning of an HTA, submit patient evidence for consideration, and be recruited as committee members to jointly develop NICE guidance and quality standards [14]. Similarly, Canada welcomes patients into its HTA process, allowing them to make submissions and participate in recommendation meetings to discuss HTA reports [15]. Empirical research has demonstrated that patient involvement can offer unique insights into technology, lead to a rephrase of findings, and enrich the content of HTA reports and recommendations [16, 17].

Some LMICs have introduced norms and infrastructure that support transparency and patient involvement [18]. Previous studies conducted in Nepal, Thailand, Brazil, China, and Bulgaria have highlighted the importance, experiences, challenges, and effects of patient involvement in HTA [19–22]. However, for many other LMICs, patient involvement in HTA remains an emerging concept [19]. To address this knowledge gap, a comprehensive overview of the research field is needed. This study applied a bibliometric analysis to summarize the current state, identify hot topics, and discuss trends in global research on patient involvement in HTA. These findings may help to inform future research for the LMICs.

## Methods

### Data source and retrieval strategy

The data were obtained from the core databases of Web of Science, which was widely used for bibliometric studies [23, 24] because of its comprehensive coverage of high-quality literature and detailed bibliographical data, including article titles, types, authors, keywords, abstracts, citations, journal names, publication years, and lists of cited references [25].

The search strategy was determined through the following steps. First, we referred to a review of patient involvement in HTA, and initially identified the search terms [16]. Next, we added relevant synonyms and phrases based on highly cited articles in the field. The search terms were then evaluated by an expert in HTA to ensure accuracy and comprehensiveness. After several rounds of searches, the terms were optimized to better cover the research topic. The search was stopped when additional terms no longer significantly increased the number of retrieved articles.

The search was executed in January 2024, covering the period from January 1, 1900–December 31, 2023. The retrieval strategy was as follows: TS = (patient) AND TS = (involv* OR participat* OR engag* OR empower* OR voice) AND TS = ("health technology assessment"OR HTA). The initial sample consisted of 393 documents.

### Screening criteria

**Fig. 1:**
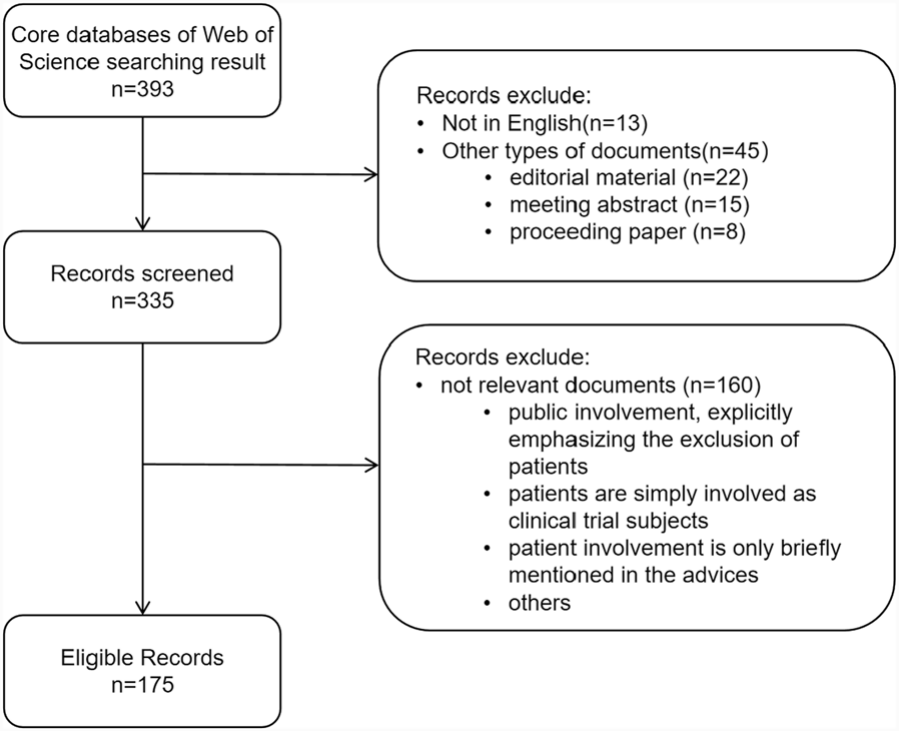
Flow chart of retrieving documents for bibliometric analysis

Figure 1 lists the inclusion and exclusion criteria. We only included English papers, and limited the document types to research articles, review articles, and early access documents. Editorial material, meeting abstracts, and proceeding papers were excluded. In addition, we screened article abstracts to identify documents related to patient involvement in HTA. Exclusions were applied when: (1) studies focused on public involvement, which did not include patients; (2) patients were simply involved as clinical trial subjects; and (3) patient involvement was only briefly mentioned in the advice.

### Validity of the search strategy

Two methods were employed to validate the search strategy. First, 50% of the articles each year were randomly selected for full-text manual review. In years with fewer than five publications, all the documents were reviewed. The high relevance of these articles indicated a low incidence of false-positive results. Second, we evaluated the false-negative results through citation analysis, utilizing the powerful functionality of the Web of Science. The top five highly cited articles were selected, and all the articles that referenced them were traced. We identified documents closely related to the research field, which were then compared with the articles retrieved via the search strategy. The search strategy captured 96.43% of the relevant articles, indicating high validity.

### Bibliometric indicators

We conducted a bibliometric analysis to uncover the current status and development trends of patient involvement in HTA research. To determine the bibliometric indicators, we referred to previous studies in fields such as health care services [26], behavioral science [27], and finance [24]. We examined the contributions of different scientific actors, and the impact of the publications, focusing on the volume of publications, the most active countries and institutions, and the most cited journals and articles. Additionally, we analyzed research themes and trends through keyword co-occurrence and burst analysis.

### Data analysis

All the valid articles were converted to VOSviewer (version 1.6.20) and CiteSpace (version 6.2. R6) to perform a visual analysis. The annual number of publications was analyzed using Microsoft Excel. VOSviewer was used for collaboration analysis (i.e., countries and institutions) and citation analysis. CiteSpace is good for detecting emerging trends within an intellectual field. It was applied to keyword co-occurrence, cluster and burst analysis. Based on knowledge domain maps, we revealed the basis, hot topics, and the evolution of patient involvement in HTA research.

## Results

### Annual number of publications

**Fig. 2:**
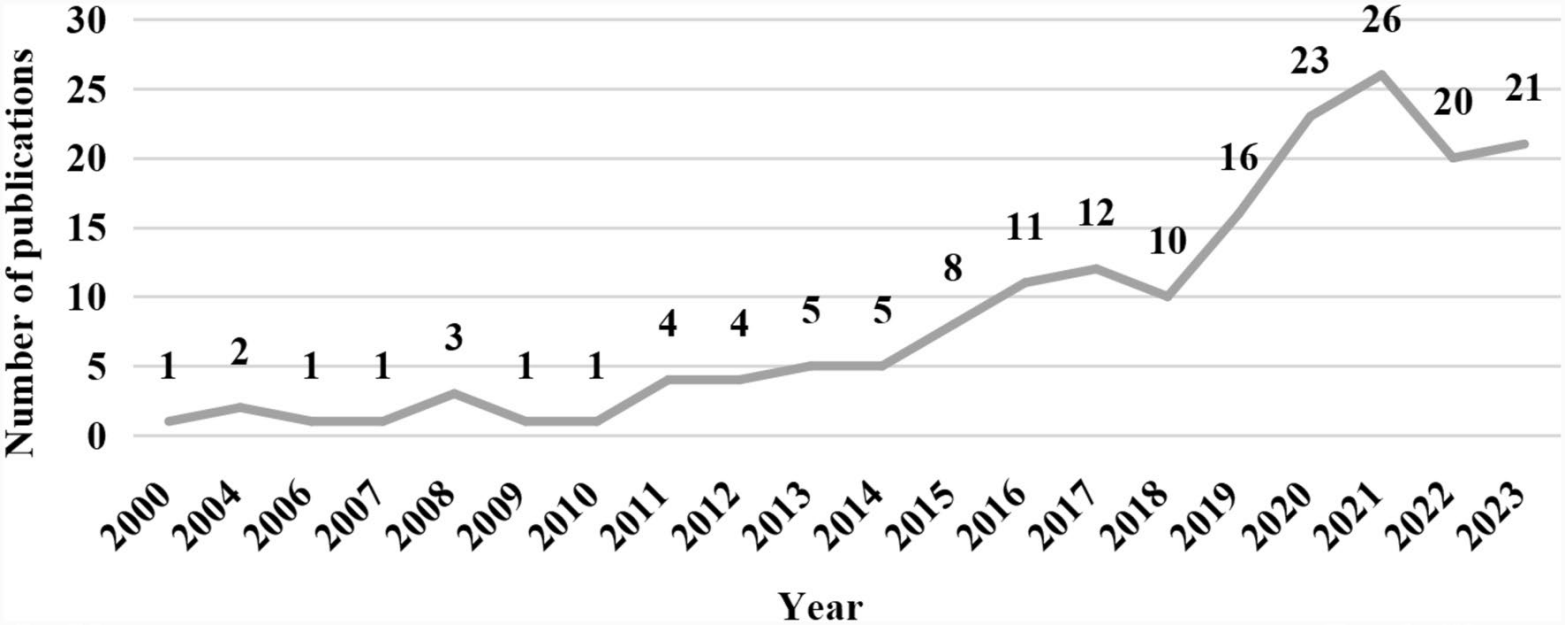
Annual number of publications on patient involvement in HTA research from 2000–2023

A total of 175 documents were eligible for this study, comprising 139 (79.43%) original research articles and 36 (20.57%) review articles. The first article included was published in 2000. The annual number of publications has shown an overall increasing trend. It has increased since 2011, peaking in 2021 at 26 articles. Approximately 80% of all the articles were published between 2016 and 2023 (Fig. 2).

### Most active countries and international collaborations

**Fig. 3:**
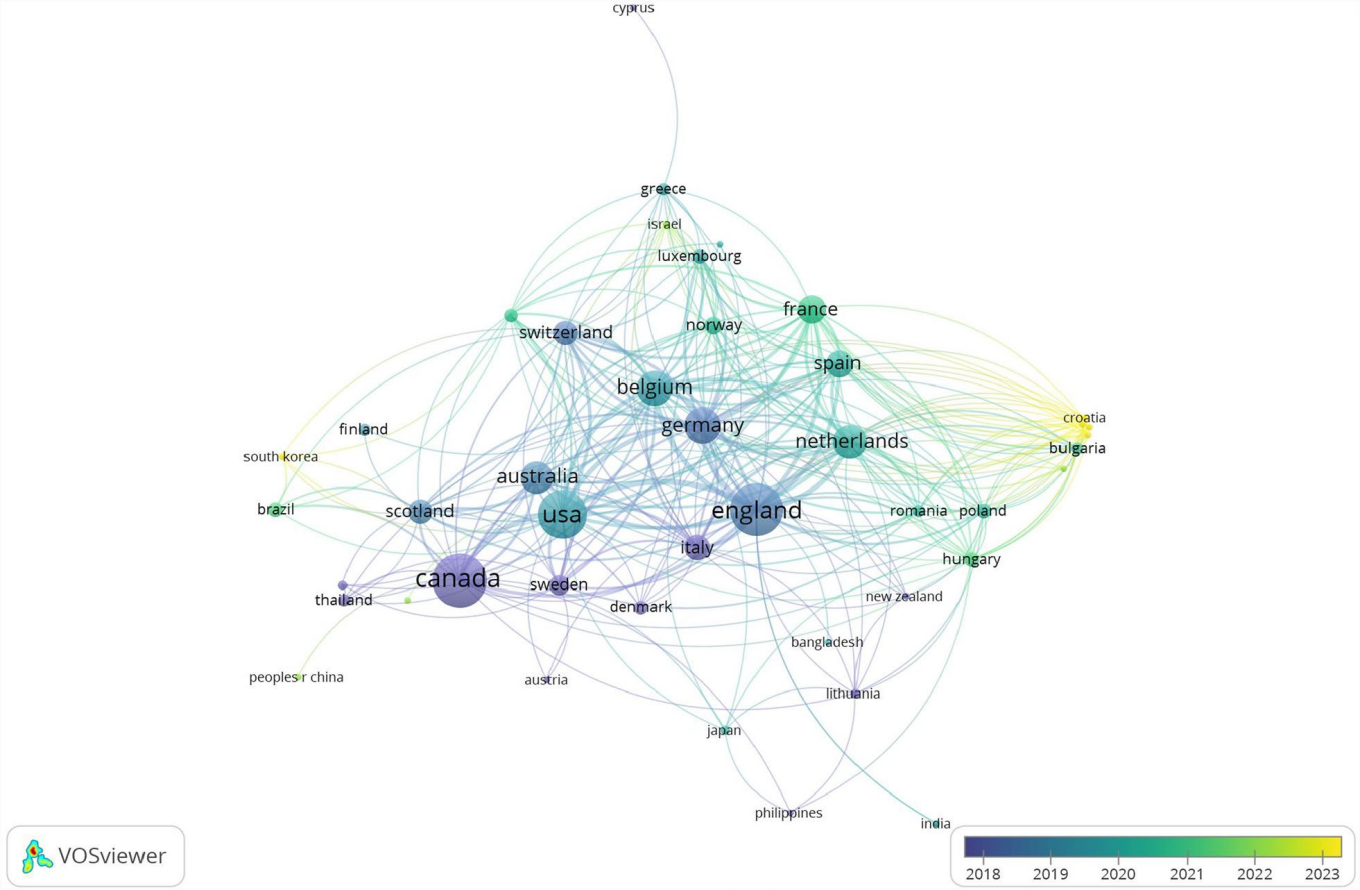
The network of national collaboration

The 175 articles came from 47 countries. The country network analysis identified eight clusters, with the majority of countries located in North America, Europe, and Oceania. Canada had the greatest number of publications (*n* = 58) and collaborated with many countries across various continents, including LMICs such as Brazil, China, Thailand, and Malaysia. England came in second place quite narrowly (*n* = 57), collaborating extensively with European countries as well as LMICs such as India and Bangladesh. Other prominent clusters were centered around the United States, Germany, and Belgium. The overlay visualization performed in VOSviewer revealed that the average publication year was concentrated between 2018 and 2023. Notably, while the participation of LMICs in publishing articles has been limited, it has gradually increased in recent years (Fig. 3).

### Most active institutions and institutional collaborations

**Fig. 4:**
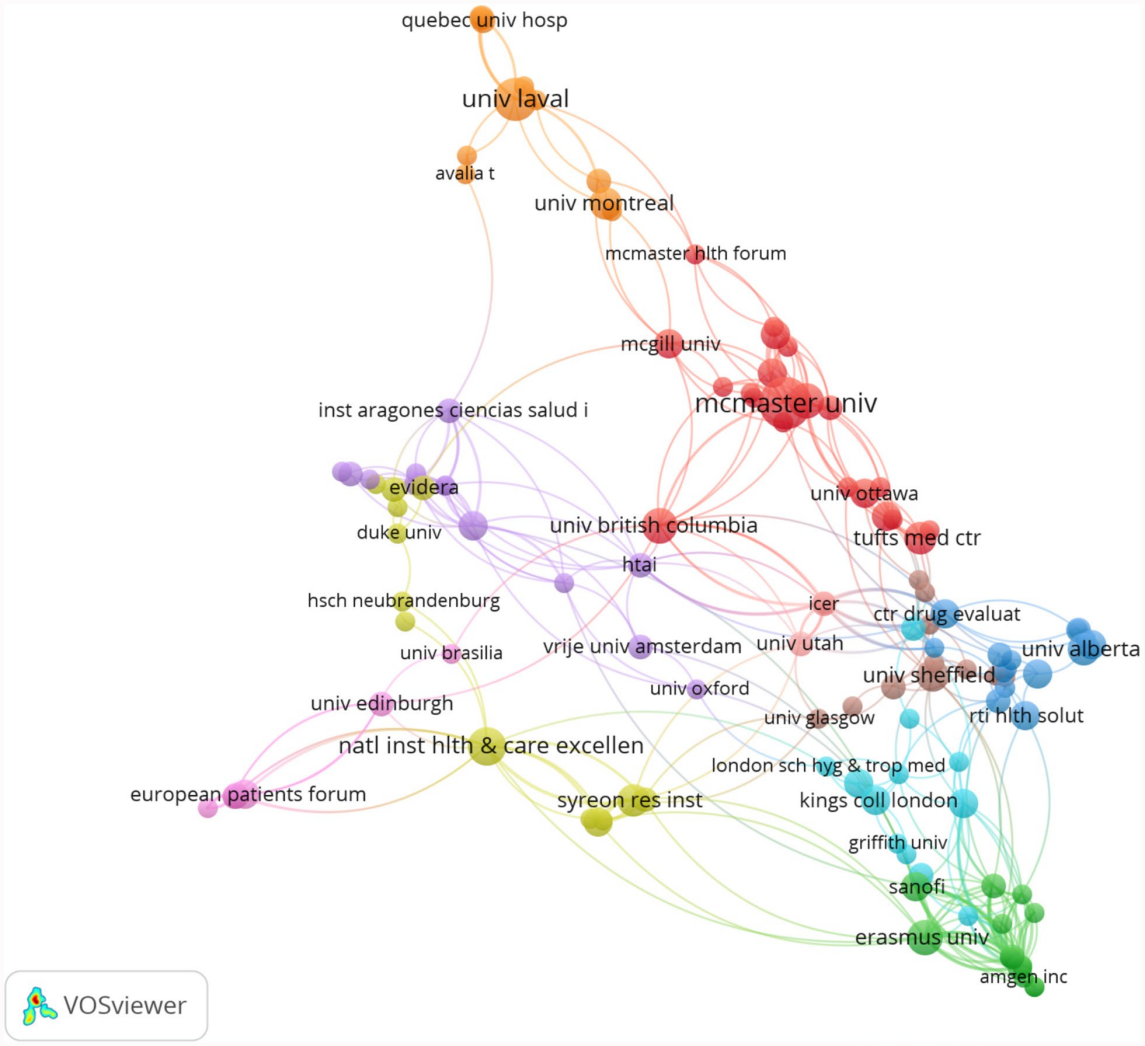
The network of institutional collaboration

A total of 556 institutions published relevant research. We selected institutions with more than two articles for mapping, which consisted of 132 institutions, mainly from North America and Europe. As shown in Fig. 4, the three most active institutions were McMaster University (*n* = 13, Canada), Laval University (*n* = 9, Canada), and NICE (*n* = 7, England). They each led some HTA bodies, universities, hospitals, and patient organizations to conduct studies, forming red, orange, and yellow clusters.

### Top-cited journals and articles: citation analysis

**Fig. 5:**
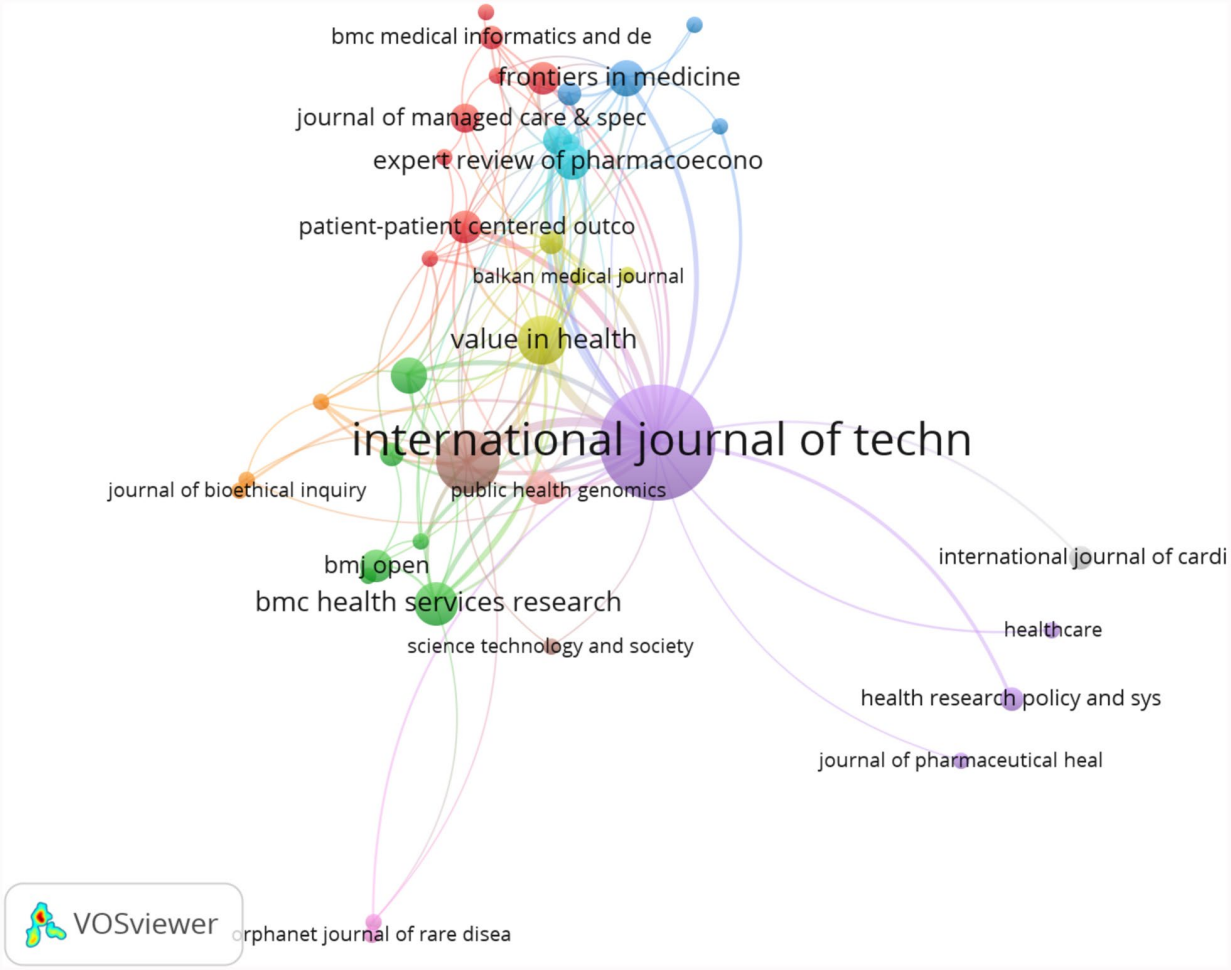
Citation network analysis of journals

A total of 13 journals have been cited more than fifty times. Figure 5 indicated that the journal with the highest number of citations was *the International Journal of Technology Assessment in Health Care* (784 citations, IF = 2.6, JCR Q2), followed by *Value in Health* (269 citations, IF = 5.6, JCR Q1), and *Health Expectations* (264 citations, If = 3.6, JCR Q2). Two of these journals are based in England, and one is based in the U.S., suggesting a strong academic presence in these regions.

The top 5 cited articles were listed in Table 1. Notably, three of the articles were published in the *International Journal of Technology Assessment in Health Care.* The most highly cited article, entitled “*Patients’ perspectives in health technology assessment: A route to robust evidence and fair deliberation”* by Karen Facey (2010), has 168 citations. This paper identified two distinct approaches to patient involvement, laying a solid foundation for further scientific research in the field. The other highly cited articles focused on international experiences, action frameworks, and methodologies for patient involvement in HTA [28].

**Table 1:** Top 5 cited articles on patient involvement in HTA research

No	Author	Title	Year	Citations
1	Karen Facey	Patients’ perspectives in health technology assessment: a route to robust evidence and fair deliberation	2010	168
2	Maria-Jose Santana	Framework to assess the effects of using patient-reported outcome measures in chronic care management	2014	138
3	Marie Pierre Gagnon	Introducing patients’ and the public’s perspectives to health technology assessment: a systematic review of international experiences	2011	105
4	Julia Abelson	Public and patient involvement in health technology assessment: a framework for action	2016	90
5	Ellen M. Janssen	Improving the quality of discrete-choice experiments in health: how can we assess validity and reliability?	2017	79

### Analysis of hot topics: keyword co-occurrence and cluster analysis

These articles included a total of 296 keywords, with twelve keywords occurring more than ten times. A keyword co-occurrence analysis performed in CiteSpace revealed that in addition to the search terms “health technology assessment” and “patient involvement”, the most frequent keywords were care (*n* = 36), decision-making (*n* = 23), framework (*n* = 19), patient preferences (*n* = 18), and perspective (*n* = 18).

A keyword cluster analysis was performed to identify the hot topics in the research field. A total of twelve clusters were formed, with S = 0.94 (> 0.7) and Q = 0.66 (> 0.3), indicating effective clustering. Table 2 revealed that in addition to the search terms, the top five research topics were patient preferences, priority setting, qualitative research, drug development, and hospital-based HTA.

**Table 2:** Key research domains of patient involvement in HTA

No	Cluster	Most frequent terms	Count	Percentage
#0	Health technology assessment	Patient participation; biomedical; gene therapy; value	45	15.2%
#1	Patient engagement	Patient involvement; patient participation; recommendations; stakeholder engagement	36	12.2%
#2	Patient preferences	Drug life cycle; health technology; patient empowerment; patient organization	23	7.7%
#3	Priority setting	Decision making; empirical ethics; deliberation; public deliberation	20	6.8%
#4	Qualitative research	Systematic review; patient perspectives; patient participation	20	6.8%
#5	Drug development	Noncommunicable diseases; comparative research; early HTA; multi-holder dialog	19	6.6%
#6	Hospital-based HTA	Patient perspective; consumer involvement; viewpoints of stakeholders; interviews	14	4.7%

### Analysis of research trends: keyword burst analysis

**Fig. 6:**
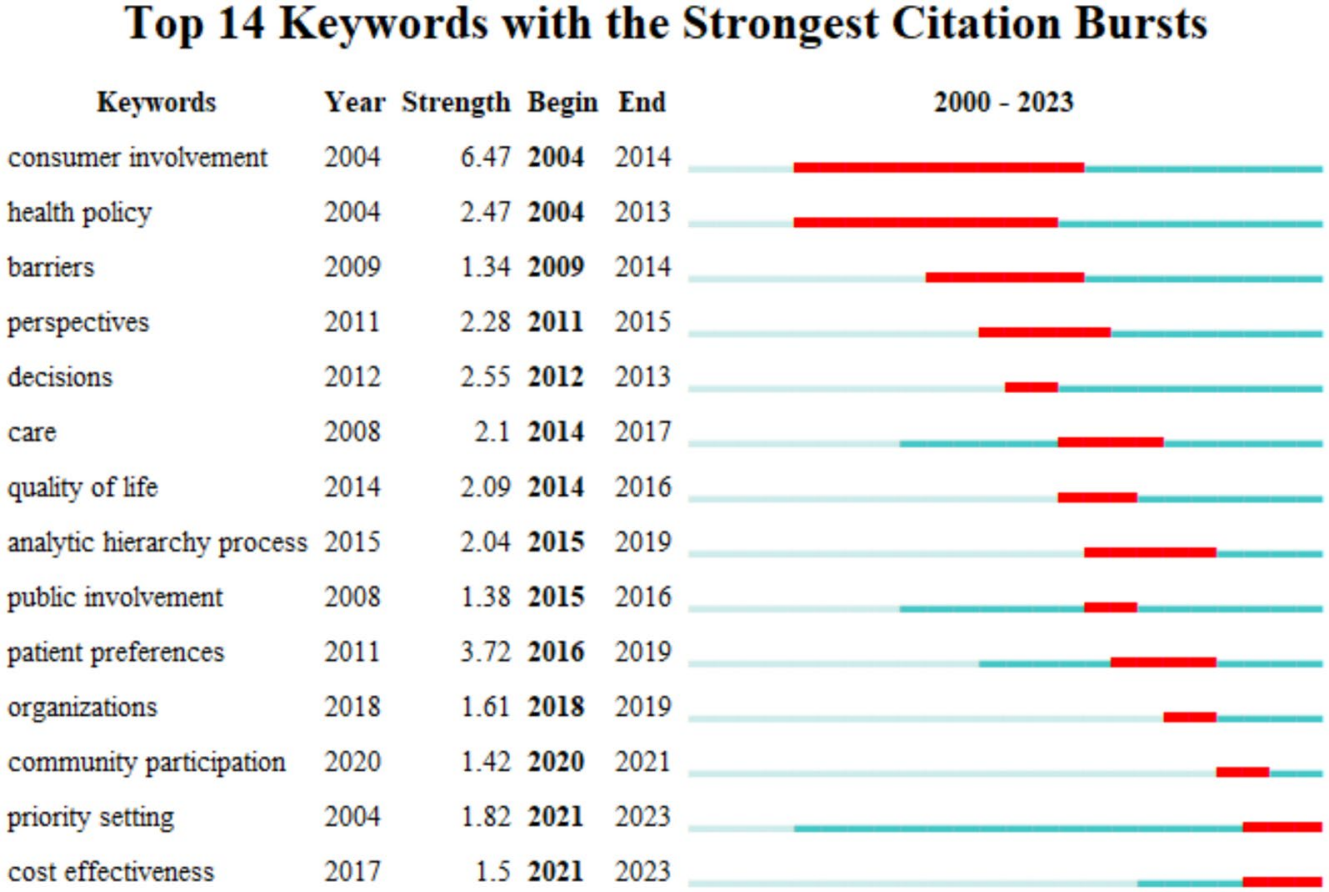
Keywords with the strongest citation bursts

A keyword burst analysis via CiteSpace was utilized to identify the research frontiers and hotspots in the corresponding period. Figure 6 presented 14 burst keywords in total. Among them, consumer involvement was the first burst keyword, with the strongest burst strength, followed by patient preferences, decisions, health policy, quality of life, etc. Priority setting and cost effectiveness have been the only two keywords that continue to be used today, which indicates there will be a growing number of relevant studies in the future.

## Discussion

In this study, we conducted a bibliometric analysis to explore the past, present, and future for patient involvement in HTA research. The growth in publications suggests that relevant research has increased since 2011, and has gained more attention in recent years. The most productive country was Canada. The journal with the highest number of citations was the *International Journal of Technology Assessment in Health Care*. Keyword co-occurrence and a cluster analysis revealed that the five hot research topics were patient preferences, priority setting, qualitative research, drug development, and hospital-based HTA. Burst analysis revealed that priority setting and cost effectiveness were the research frontiers. The findings of the study were discussed below.

### Growth of publications

The rise in publications since 2011 can be attributed to several key factors. First, some countries involved patients in the HTA process through the establishment of laws and national programs, which led to an increase in publications. For example, in 2010, the U.S. enacted the Patient Protection and Affordable Care Act, which proposed the concept of patient-centered care, and incorporated patient perspectives in comparative effectiveness research [29]. Canada initiated the patient input program as part of the common drug review process [30]. Second, increased advocacy from patient organizations heightened interest from institutions and researchers. For example, patient organizations strongly called for support for their involvement in HTA in a seminar hosted by the European Patients’ Forum (EPF) in 2010. The EPF subsequently conducted research involving 23 European countries and 50 HTA agencies. In addition, a global push for international collaboration in HTA has been underway since 2010. The European Network for Health Technology Assessment (EUnetHTA) launched Joint Action 1, which strengthened cooperation between the HTA participants (including patients) in Europe in practice and research [31]. Furthermore, the HTA Core Model has explicitly included patient and societal aspects since 2016, providing standardized tools for HTA across countries, which resulted in a rapid development phase for the research field [32].

### International and institutional collaboration

International collaboration on patient involvement in HTA research has increased notably, with Canada and England leading in publication output, and acting as key centers for global collaboration. This aligned closely with the advanced development of HTA. Both countries introduced HTA in the 1990 s, and were early adopters of patient involvement [8].

In terms of collaboration patterns, the clusters led by Canada and England exhibited some differences. Canada collaborated widely across Europe, Oceania, Asia, and South America, including several LMICs. Canadian institutional collaborations are primarily university-led, partnering with geographically proximate universities, HTA agencies, and medical institutions. England primarily collaborated with countries in Europe and North America, with the HTA body “NICE” at the center of the cluster. The committees of the NICE include various stakeholders, such as patient organizations, universities, and pharmaceutical companies, providing a strong foundation for collaboration. Both countries have research collaborations with the LMICs, facilitating the ability of the LMICs to increase awareness and further their research on patient involvement.

International and institutional collaboration promoted the dissemination of the concept of patient involvement, and significantly increased the publication output in the European Union, North America, and Oceania. Moreover, researchers and evaluators were able to exchange insights and identify strengths and challenges within their respective regions. This cooperation has played a crucial role in advancing local and global studies and practices.

### Journals

Our results indicated that the journal with the highest number of citations was *the International Journal of Technology Assessment in Health Care*, which was notable for its high impact articles. Although it was not the journal with the highest JCR region and IF, it had expertise and authority in the field of HTA and was highly recognized by the academic community. The articles it published provided important references and guidance for researchers in the field. Furthermore, *Value in Health* and *Health Expectations* published high-quality research articles as well. Researchers can consider these priority journals in the future to gain more attention from the academic community.

### Five hot topics

The first hot research topic was patient preferences. Studies have emphasized the importance of patient preferences in HTA. Patients’ views are often different from those of other stakeholders, and the inclusion of patient preferences helps to better understand patient value from a health condition perspective, and improves patients’ adherence and satisfaction with health technologies [33, 34]. Previous studies focused on HTA stages that can incorporate patient preferences, such as identifying the ‘right’ issues [33] and constructing and evaluating value frameworks [35]. Some scholars have also explored the integration of patient preferences into the life cycle of drug development [36]. Other studies have focused mainly on methodologies, such as how to quantify patient preferences, and have assessed the validity and reliability of these methods [37]. However, scholars have proposed that how to incorporate patient preferences in HTA systematically and scientifically needs to be further discussed [34].

The second hot research topic was priority setting. It is a term commonly used in health economics and HTA. In the context of limited budgets and existing resource allocations, priority setting is used to determine who obtains what (e.g., technologies). Recent studies have demonstrated that public (including patient) values are influential to priority-setting practices and frameworks [38]. For some treatments that currently lack the formal inclusion of cost-effectiveness, patient values are critical to their reimbursement policy [39]. Scholars have shown great interest in how patients can be understood and supported in relation to priority setting [40]. One main approach was encouraging patients to listen, express, and discuss their views through deliberation, forming a commonly agreed-upon set of outputs that could be used for policy decision-making [38, 39].

The third hot research topic was qualitative research. Qualitative methods can generate patient-focused evidence relevant to HTA, which is complementary to studies of clinical and cost-effectiveness [41]. A large share of existing studies were systematic reviews that focused on aggregating various evidence about patients’ perspectives, such as views, experiences, and preferences [28]. In addition, some studies applied a series of interviews or focus group discussions with different stakeholders, which helped elicit patients’ perspectives and uncover topics not identified beforehand [42, 43]. Ethnographic analysis was also used to analyze the ways and problems of patient involvement in HTA processes [44]. Improving the quality of qualitative research and innovating qualitative methods are future research priorities [41].

The fourth hot research topic was drug development. A growing number of articles have demonstrated that patient perspectives are essential for all levels of decision-making throughout the life cycle of drugs [36]. Previous studies have noted that introducing patient preferences in early drug development can inform patient-reported outcome strategies, clinical development strategies, and product design [45, 46]. It can also form the basis for an early dialogue with multiple stakeholders, including regulators, HTA bodies, and payers, to ensure a focus on patient-relevant endpoints [45]. Additionally, some scholars have applied multicriteria decision analysis to conduct early HTA during new drug development [47]. Notably, many of these studies have concentrated on drugs for noncommunicable diseases, particularly rare diseases [48] and oncological diseases [49].

The fifth research topic was hospital-based HTA. Introducing patient involvement in hospital-based HTA provides opportunities to foster patient involvement in decisions at the local level [10]. Most of the studies were conducted in Canada. Previous studies have explored how local HTA stakeholders perceive patient involvement in hospital-based HTA, including its relevance, feasibility, barriers, and facilitators, and the best ways to introduce patients’ perspectives [10, 50, 51]. Although patient involvement in hospital-based HTA has received increasing attention in recent years, it has not been widely implemented and studied.

### Research trends and frontiers

Over the more than 20 years of research on patient involvement in HTA, researchers have gradually clarified the properties of the participants (on behalf of patient’s benefits) in HTA. During the early years (2004–2014), researchers typically used ‘consumers’ to represent these participants. Later, studies used ‘public’, ‘patient’, ‘organizations’, and ‘community’ to denote such participants. Each term represented a different scope of people, and contributed diverse patient perspectives. Since 2015, methodological research on how to access and understand various types of patient evidence (especially patient preferences) developed rapidly, including the development of scales to measure patients’ quality of life, analytic hierarchy processes, etc. Currently, the research frontier concentrates on setting priorities by introducing a wide range of ‘patients’ and adopting innovative research methods. As discussed earlier, when a health technology has limited economic evidence, or is close to/exceeding the cost-effective threshold, introducing patient involvement to supplement the assessment evidence and elucidate the unintended or indirect impacts on patient outcomes becomes more critical to decision-making.

### Challenges and Implications for LMICs

Our findings revealed that publications conducted in LMICs were significantly fewer than those conducted in high-income countries. There are some factors that may contribute to this discrepancy. First, HTA-related research in LMICs began later, with a focus on clinical effectiveness and cost effectiveness, and patient aspects that aimed to provide complementary evidence were given a lower priority. Second, in high-income countries, institutional support and funding from governments have accelerated research development, whereas in the LMICs, few policies exist, and researchers have limited understanding about patient involvement.

The implementation of patient involvement in LMICs faces complex challenges, including policy, resources, culture, and social contexts. First, some LMICs have not included HTA in decision-making, and only a few have established policies, mechanisms, or guidelines on patient involvement, which require further improvement [52]. For example, in Brazil, although public involvement in HTA is mandatory, patients mainly participate at the consultation level, and the decision-making process still lacks sufficient transparency [53]. Second, introducing patient involvement requires payers, HTA bodies and patient organizations to input considerable amounts of time, human resources and funds, which are often limited in LMICs [19, 22]. This hinders the recruitment, training, and engagement of patients in the HTA process [22]. Cultural and social factors also play a significant role, with patient participation rates varying by ethnicity, education, and economic status, as seen in Brazil [54]. In Nepal and Bangladesh, the dominance of health workers in decision-making limits both patients’ willingness to be involved and their decision-making influence [19].

Addressing these challenges requires collaboration among policy-makers, patient organizations, and researchers. Policy-makers should recognize the value of patient involvement by learning from international experiences, and enhance policy translation. It is recommended that policies or plans for patient involvement be established, and that specific participation stages be clarified. For example, patient representatives can be permitted to join committees [21, 55], participate in health topic nominations [20], submit perspectives and evidence for assessment [22], attend meetings [22], provide comments to HTA reports [22], and offer opinions on recommendations [21, 22]. This allows for the early and comprehensive inclusion of patients in the HTA process, thereby enhancing transparency in decision-making. In LMICs where HTA is in the early stages, introducing patient involvement from the beginning may be a good choice [14]. However, challenges remain, including a potential lack of political will to involve people other than government officials and experts in decision-making, as well as insufficient support for the human, financial, and technical resources.

For patient organizations, a key strategy is to strengthen their impact. In some LMICs, inviting leaders from religious communities or civil society can increase the social recognition of these organizations [19]. Collaboration with the academic community is also crucial. Patient organizations can collect and provide information on patient demographics, disease conditions, medications, treatments, and actual outcomes for research. Disseminating this research can enhance the influence of patient organizations, particularly in countries where experts dominate the decision-making. Leaders should receive professional training and educate their members to improve the awareness and competence in their own involvement. Patient organizations should also actively communicate with government departments to advocate for participation in the decision-making process, and gain policy support. However, patient capacity remains limited in the LMICs [20, 22, 55], and building this capacity requires a long-term process.

Researchers should improve their research capacity and impact by actively engaging in international collaboration. Joining organizations such as HTAi and INAHTA, and regional networks such as HTAsiaLink (Asia) and RedETSA (Latin America), is recommended. Strengthening cooperation with governments, HTA bodies, universities, and pharmaceutical companies in high-output countries (e.g., Canada and the UK) can enhance research influence both domestically and internationally. In addition, researchers can deepen their understanding of the field by studying highly cited journals, carrying out adequate research on the five hot research topics, and focusing on emerging frontiers. The most cited journals can be the journals that researchers prioritize. Concurrently, the alignment of localized research with domestic practices will help researchers gain unique insights. Additionally, researchers should advocate for patient involvement to help policy-makers and health workers better consider patient needs and perspectives in decision-making.

In the long term, LMICs need to address resource shortages and limited patient capacity. Governments and HTA organizations can create dedicated funds to support the local academic community, which in turn facilitates the investment of limited resources in the areas that will most significantly improve patient benefits. Recruiting and training more professionals is essential for strengthening patient organizational capacity. This can be achieved through regular training, expert lectures, and research on international guidelines. Effective patient involvement in HTA can also be supported by technical assistance. For example, constructing online platforms can expand the channels for patients to submit evidence and express their opinions.

### Strengths and limitations

To the best of our knowledge, this is the first study to perform a bibliometric analysis on patient involvement in HTA research, and it describes the distribution, hot topics, frontiers, and trends in this field. However, this study has several limitations. First, we only searched for articles from the core databases of Web of Science, potentially excluding some relevant literature from other important databases. Despite this, we believe that the Web of Science database contains enough data for a bibliometric analysis, and that our principal findings are robust. A broader range of databases could be used in the future to further validate our findings. Second, the frontiers and trends were identified through a burst analysis, and it is necessary to follow them up continuously.

## Conclusions

Patient involvement in HTA research is gaining increased attention. However, studies conducted in the LMICs are still limited. In the future, it is recommended that the LMICs join in international research collaborations and explore the hot research topics and emerging frontiers summarized above. Based on the experiences of the high-income countries, policy-makers, and patient organizations, researchers in the LMICs should work together to advance both their research capacity and practical implementations.

## Data Availability

The datasets used and/or analyzed during the current study available from the corresponding author on reasonable request.

## Abbreviations

HTA: Health technology assessment
LMICs: Low- and middle-income countries
NICE: National institute for health and care excellence
JCR: Journal citation reports
IF: Impact factor
HTAi: Health technology assessment international
INAHTA: International network of agencies for health technology assessment
